# Circuit-based neuromodulation for obsessive-compulsive disorder: a review of prior and emerging methods

**DOI:** 10.3389/fpsyt.2026.1828620

**Published:** 2026-07-13

**Authors:** Peter M. Lauro, Charles F. Palmer, Marshall M. Nambiar, Ully Muller, Liming Qiu, Casey H. Halpern, Katherine W. Scangos

**Affiliations:** 1 Department of Psychiatry, University of Pennsylvania, Philadelphia, PA, United States; 2 Department of Neurology, University of Pennsylvania, Philadelphia, PA, United States; 3 Department of Neurosurgery, University of Pennsylvania, Philadelphia, PA, United States; 4 Independent Researcher, Rio de Janeiro, Brazil

**Keywords:** deep brain stimulation, focused ultrasound, neuromodulation, obsessive-compulsive disorder, psychiatric neurosurgery, transcranial magnetic stimulation

## Abstract

Obsessive-compulsive disorder (OCD) is a psychiatric disorder characterized by intrusive and distressing thoughts (obsessions) and repetitive/ritualistic behaviors (compulsions). OCD pathophysiology is thought to arise from dysregulated cortico-striato-thalamo-cortical (CSTC) loops between cognitive and affective circuits. Clinically effective neurosurgical lesions such as cingulotomy and capsulotomy provide insight into mapping distinct symptoms to circuits, and more modern neuromodulation techniques such as transcranial magnetic stimulation (TMS), deep brain stimulation (DBS), and focused ultrasound (FUS) provide the opportunity to develop more precise interventions. We review the literature for neuromodulatory interventions for OCD with a focus on identifying common circuits across modalities. Future directions such as closed-loop DBS and more precisely identifying symptom subtypes are also discussed.

## 1 Introduction

**Figure 1:**
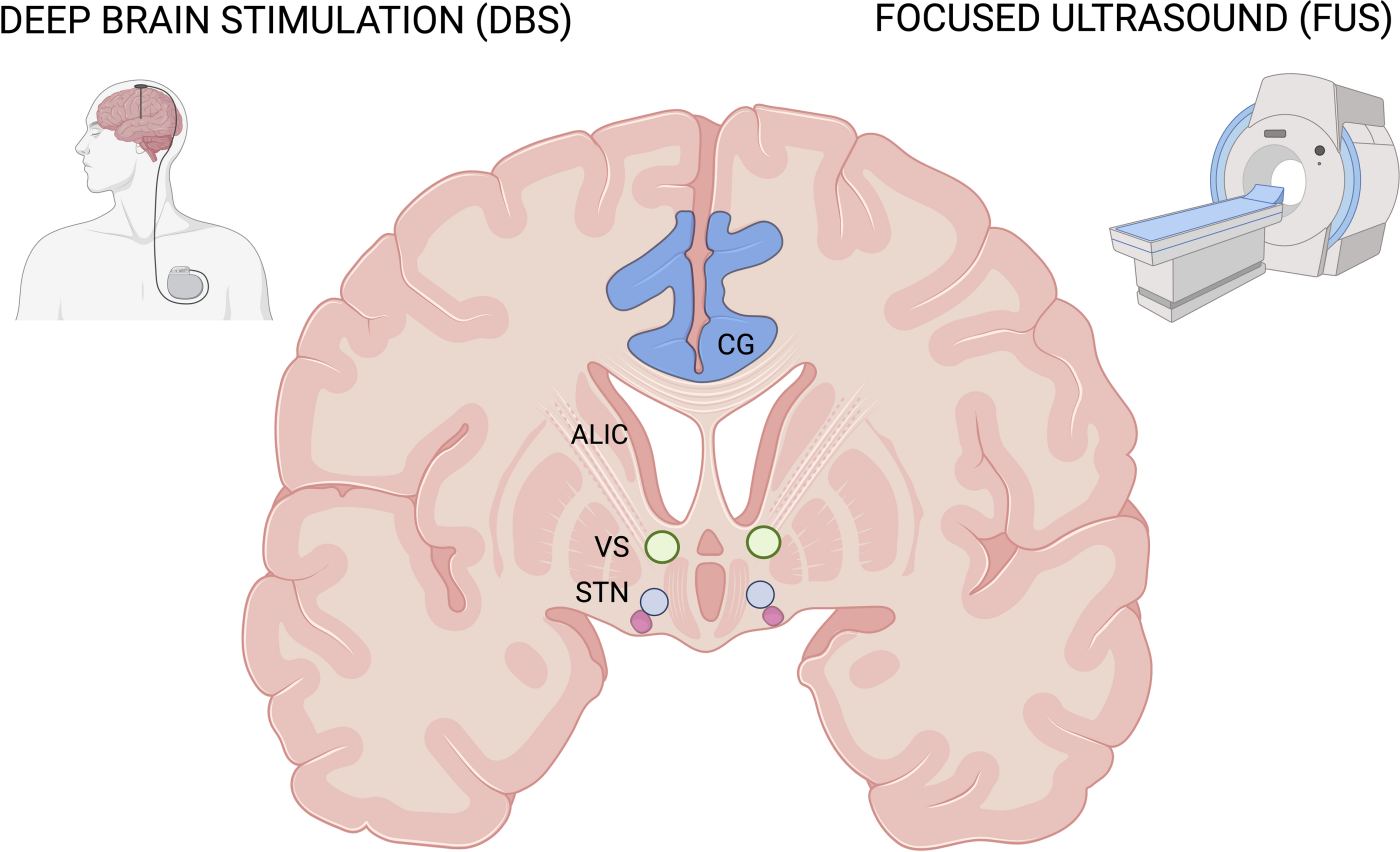
Superficial TMS targets and subcortical areas thought to be affected by TMS.

**Figure 2:**
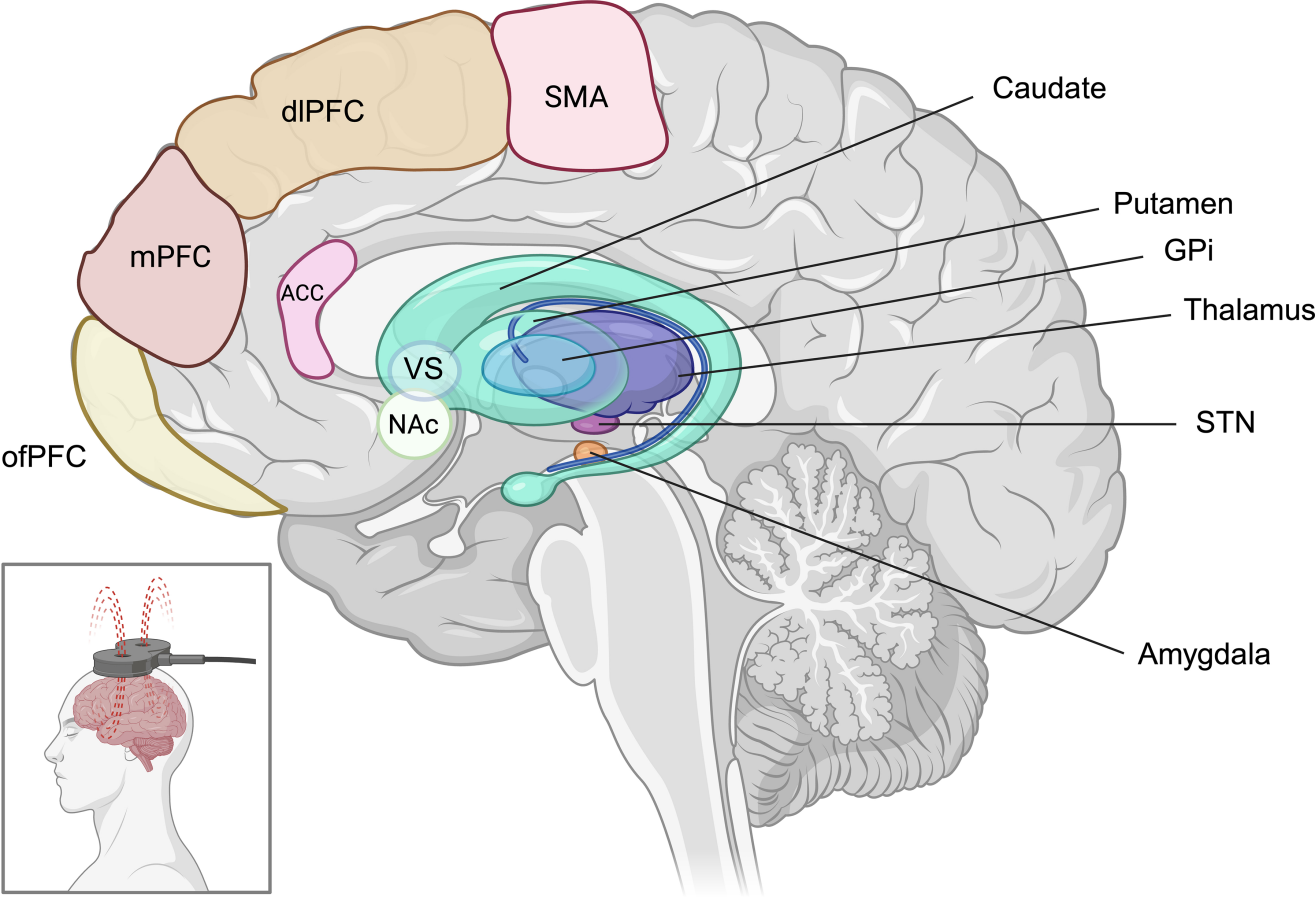
Deep targets accessible by neurosurgical lesions, deep brain stimulation (DBS), and focused ultrasound (FUS).

Obsessive-compulsive disorder (OCD) is a psychiatric disorder characterized by intrusive and distressing thoughts, mental images, impulses, or urges (obsessions) and repetitive/ritualistic behaviors (compulsions) which affects 2-3% of the US population (1, 2). Although obsessive-compulsive phenomena were initially conceptualized psychodynamically as defenses against intra-psychic conflict between desires and inhibitions, it is increasingly understood as a dysregulation of multiple cognitive and affective circuits (3, 4). Despite first-line therapies such as cognitive behavioral therapy with exposure and response prevention [CBT-ExRP (5)] and serotonin reuptake inhibitors [SRIs (6–8)], 30-40% of patients fail to respond (9, 10). As refractory OCD can lead to functional impairment and increased mortality, there remains an unmet need for additional treatment modalities (11, 12). With this challenge comes the opportunity to use non-invasive (transcranial magnetic stimulation – TMS, focused ultrasound – FUS; **Figure 1**) and invasive (deep brain stimulation – DBS, neurosurgical lesions; **Figure 2**) neuromodulation to probe and leverage the neurocircuitry of OCD. In this review, we will bridge mechanistic insights from neurosurgical procedures to present conceptions of circuit-based OCD pathophysiology, with a focus on emerging treatment approaches.

## 2 Anatomical and functional neurocircuitry of OCD

From a neurobiological perspective, converging lines of evidence suggest that OCD psychopathology arises from dysregulated cortico-striato-thalamo-cortical (CSTC) loops (13, 14). Positron emission imaging (PET) and functional magnetic resonance imaging (fMRI) studies have implicated several key brain regions (orbitofrontal cortex – OFC, anterior cingulate cortex – ACC, caudate, ventral striatum – VS) which demonstrate altered activity in response to OCD symptom provocations (15–17). As these regions are involved in neural networks mediating the balance between goal-directed behavior and habit-learning systems, core features of OCD such as harm avoidance (active avoidance of negative outcomes) and incompleteness (requirement of thought/action perfection before proceeding) may reflect disorders of learning, cognitive control, and action selection (1, 18). Furthermore, hypothesized increased activity in the direct striatonigral pathway (relative to indirect striatopallidal) acts upon these CSTC loops to inadequately filter intrusive thoughts and promote repetitive behavior (19).

Advances in characterizing psychiatric symptoms with neuroimaging (20) and electrophysiology (21) suggest a shift towards mapping symptoms to circuits rather than targets (4, 22). For example, whereas intolerance of uncertainty is hypothesized to be mediated by a frontolimbic circuit (amygdala-vmPFC), other aspects of OCD such as impaired reward learning and emotion regulation are respectively seated in ventral affective (VS-OFC) and dorsal cognitive (caudate-dlPFC) circuits. Although retrospective analyses of multiple DBS targets (anterior limb of the internal capsule – ALIC, VS, anteromedial subthalamic nucleus – amSTN, ventral tegmental area – VTA) have suggested common therapeutic white matter streamlines, detailed anatomical studies in non-human primates have revealed more distinct circuits at each target (20, 23). As neuromodulation efforts in OCD have focused primarily on global symptom improvement as measured by the Yale-Brown Obsessive-Compulsive Scale (YBOCS) rather than focusing on specific symptom dimensions (mood, inhibitory control, approach-avoidance behavior), directly testing the relationship between circuits and symptoms remains largely unexplored (24).

Imaging studies often provide static (structural connectivity) or temporally coarse (PET, fMRI) representations of underlying neurocircuitry. However, it is now possible and increasingly common to record from *in vivo* brain structures in the medical setting to more precisely characterize functional correlates of OCD pathophysiology. Efforts towards closed-loop or adaptive deep brain stimulation (DBS) within psychiatry [OCD (25), depression (26, 27)] and neurology [Parkinson’s disease (28, 29)] have revealed that symptom states and their associated neural biomarkers often fluctuate on short timescales. Moving forward, neuromodulation with the dual aim of research and clinical care offers the unique promise to probe and hypothesis test the underlying function of neural circuits. OCD pathophysiology in particular demonstrates interactions between cognitive, affective, and motor circuits which may yield insights across the neuropsychiatric disease spectrum.

In this narrative review, we first discuss the lessons learned from lesional therapies, which predate the advent of neuromodulatory techniques but remain relevant today. Based on the effects from this early work, we then describe the invasive, noninvasive, and emerging techniques currently used to modulate the OCD network. To generate the literature for this review, we searched PubMed and Google Scholar using the terms obsessive compulsive disorder, circuit, mechanism, capsulotomy, cingulotomy, deep brain stimulation, transcranial magnetic stimulation, focused ultrasound, transcranial direct current stimulation, electroconvulsive therapy. Although no formal inclusion/exclusion criteria were applied, resulting articles were selected based on their relevance to our question of identifying common neural circuits throughout neuromodulation modalities. We also emphasized articles including randomized control trials (sham-controlled when appropriate) when describing clinical efficacy. Throughout this review we define clinical response as a ≥ 35% reduction in YBOCS and “improvement” as any reduction in YBOCS.

## 3 Circuit insights from lesion studies

### 3.1 Cingulotomy

Anterior cingulotomy involves creating bilateral lesions in the cingulum bundle with the hypothesized effect of disconnecting limbic structures from frontal cortex (relevant studies listed in **Table 1**) (30). An early case series of 32 patients with intractable OCD undergoing cingulotomy from 1962–1982 demonstrated that 56% of patients were functionally well or improved, with follow-up neuropsychological testing decades later revealing no obvious deficits (31, 32). Later prospective studies on 64 patients with intractable OCD receiving capsulotomies between 1989–2009 revealed that 47% and 22% of patients had full or partial responses respectively at a mean follow-up at 64 months (33). However, this clinical improvement appeared to come at the cost of reduced behavioral spontaneity (34).

**Table 1:** Studies of bilateral anterior cingulotomy for refractory OCD.

Study	Design	Target/protocol	N	Response	Main findings
Ballantine 1987 (31)	Retrospective case series, 1962–1982	Bilateral anterior cingulotomy, thermal radiofrequency	32	56% functionally well or improved per 0–5 subjective scale	First large cohort showing benefit; basis for modern cingulotomy practice
Sheth 2013 (33)	Prospective long-term cohort, 1989–2009	Bilateral RF cingulotomy	64	47% full (≥ 35% YBOCS reduction) + 22% partial response (25-34% reduction) at mean 64-mo follow-up	Largest prospective cingulotomy series for refractory OCD

Research studies using awake microelectrode recordings in patients with OCD undergoing cingulotomy find that neural firing in cingulate cortex modulates with conflict tasks such as the Stroop, while also incorporating predictions of expected cognitive demand to modulate reaction times (35, 36). As this ability to adapt is abolished after surgery, it was hypothesized that cingulotomy may provide symptom relief by abolishing aberrant estimations of conflict. Retrospective analyses of cingulotomy lesion location suggest more superior and posterior lesions are more effective, with clinical response correlating with lesion overlap with functional ventral attention networks, but not by specific white matter bundles (37, 38). Taken together, it appears that cingulotomy may work to stop propagation of incorrect prediction errors and/or the inability to adapt estimations of control (broadly encapsulating the incompleteness dimension of OCD throughout fronto-subcortical circuits) (39). More simply, cingulotomy may restore adaptive cognitive control and allow patients to exit distressing obsession-compulsion loops (40, 41).

### 3.2 Capsulotomy

Anterior capsulotomy describes placing lesions in the anterior limb of the internal capsule (ALIC) with the aim of disconnecting fibers between prefrontal cortex, thalamus, and striatum (relevant studies listed in **Table 2**) (42). Although a long-term study (mean follow-up 10.9 years) of 25 patients receiving capsulotomy from 1988–2000 demonstrated a 48% responder rate, 50% of patients experienced apathy and impaired executive function (43). To overcome these drawbacks, a clinical trial of stereotactic radiosurgery (gamma knife) which refined lesion placement more ventrally resulted in 56% of patients demonstrating a response in YBOCS scores with minimal apathy (1/55 patients) and improvement on executive functioning (44, 45). Due to concerns of unpredictable radiation-induced volumes, ventral capsulotomy lesions have since been replicated by MR-guided laser thermal ablation, with 78% of patients demonstrating YBOCS response in one case series of 9 patients (46). Regardless of surgical technique, a large retrospective study of 70 patients undergoing capsulotomy revealed that ~13% of patients exhibited weight gain (although precise measurements are often lacking) (47).

**Table 2:** Studies of anterior capsulotomy (radiofrequency, gamma knife, laser thermal) for refractory OCD.

Study	Design	Target/protocol	N	Response	Main findings
Rück 2008 Long-term capsulotomy cohort (43)	Prospective, mean 10.9-yr follow-up (1988-2000)	Radiofrequency anterior capsulotomy (ALIC)	25	48% response	50% with apathy/impaired executive function
Lopes 2014 (44)	Double-blind RCT (8 active: 8 sham)	Gamma-knife ventral capsulotomy	16	Active arm responders > sham	Only randomized sham-controlled lesional trial for OCD
Rasmussen 2018 Gamma-knife evolution series (45)	Pooled clinical series	Gamma-knife ventral capsulotomy	55	56% response	Only 1/55 with apathy; ventral targeting preserves/improves executive function
McLaughlin 2021 (MRgLITT) (46)	Open-label case series	MR-guided laser thermal ablation, ventral capsulotomy	9	7/9 (78%) response	LITT permits larger, demarcated lesions with real-time MRI feedback

Post-capsulotomy studies investigating resting-state fMRI before and after capsulotomy found that reduction in connectivity between VS/nucleus accumbens (VS/NAc) and dorsal anterior cingulate (dACC) correlated with YBOCS improvement (48). Moreover, preoperative connectivity between dorsal caudate and dACC distinguished clinical responders from nonresponders. Other studies investigating structural connectivity with diffusion tensor imaging (DTI) before and after capsulotomy found that lesions placed more ventrally and intersecting with OFC streamlines were more associated with clinical response (49).

More nuanced work combining fMRI and neuropsychological testing found that post-capsulotomy OCD patients demonstrate BOLD decreases in nucleus accumbens (NAc) during negative anticipation and left rostral cingulate/inferior frontal cortex during negative feedback, suggesting capsulotomy may interfere with pathologically active negative emotion processing (harm avoidance) (50). In summary capsulotomy appears to blunt extreme negative affect, which may remove an obstacle for effective extinction learning (51).

## 4 Leveraging circuits for neuromodulation

### 4.1 Deep brain stimulation

Based on the earlier efficacy of cingulotomy and capsulotomy, DBS was first used in 1999 for OCD due to its proposed mechanism of creating a “functional lesion” (52) (relevant studies listed in **Table 3**). Subsequent work has suggested that DBS is not merely due to a lesional effect, but can lead to network-wide modulation over a time period of months to years. DBS involves the placement of stimulating electrodes (typically at 130 Hz) in deep brain structures. Initial studies investigating DBS of the ALIC led to FDA Humanitarian Device Exemption approval for ventral ALIC DBS for OCD in 2009 (52–54). Ongoing research has investigated other structures, including the ventral striatum/nucleus accumbens (VS/NAc) (55, 56), subthalamic nucleus (STN) (57), bed nucleus of stria terminalis (BNST) (58, 59), anteromedial globus pallidus (amGPi) (60), superolateral branch of the medial forebrain bundle (slMFB) (61), and the inferior thalamic peduncle (ITP) (62, 63). Across studies, patients demonstrated YBOCS response rates as high as 66% at last follow-up (64). Although the relative expense and follow-up burden can act as barriers towards wider use, DBS provides similar clinical efficacy to neurosurgical lesions with additional benefits of reversibility and programmability (65–67).

**Table 3:** DBS studies for OCD across stimulation targets..

Target	Study	Design	Parameters	N	Response	Main findings
ALIC	Nuttin 1999 (52)	First-in-human open-label	Bilateral ALIC DBS, 100 Hz	4	3/4 with clinical benefit; one with >90% reduction (subjective reporting)	Established DBS for psychiatric illness; basis for FDA HDE 2009
VC/VS	Greenberg 2010 (53)	Multi-center open-label	Bilateral VC/VS DBS, 100–130 Hz	26	61.5% response rate at last-follow (3–36 months)	Outcomes improved as target was refined more posteriorly to junction of capsule, anterior commissure, and posterior VS
Anteromedial STN	Mallet 2008 (57)	10-mo double-blind crossover RCT	Bilateral amSTN DBS, 130 Hz, two 3-mo on/off periods	16	75% response rate (>25% YBOCS reduction) in active, 38% with sham	Level I evidence for amSTN DBS; symptoms relapsed in OFF period; 4 surgical SAEs
BNST/IC-BST	Luyten 2016 (58)	Long-term cohort with ON/OFF crossover	Bilateral IC/BST DBS, 130 Hz	24	67% response rate	Stimulation (not implantation) drives benefit; BNST may outperform ALIC
amGPi	Nair 2014 (60)	Case series	Bilateral amGPi DBS, 120–160 Hz	4	Improvement in obsessive compulsive inventory, Yale global tic severity scale	Limbic pallidal target useful in OCD ± Tourette’s
slMFB	Coenen 2017 (61)	Case series	Bilateral slMFB DBS, 130 Hz	2	100% response rate at 1 year	Reward-circuit-based target
ITP	Jiménez-Ponce 2013 (62)	Open-label phase-I, 2003–2007	Bilateral ITP DBS, 130 Hz	5	51% YBOCS reduction at 1 yr	Connects OFC with thalamus
VC/VS vs STN	Tyagi 2019 (69)	Within-subject comparison	12 weeks of counterbalanced VC/VS or amSTN DBS, 130 Hz		50% response rate after amSTN, 83% response after VC/VS, 100% response with optimized stim at both targets	VC/VS → mood (MADRS); STN → cognitive flexibility (set-shifting)

Recently, analysis of DTI tractography across multiple different DBS targets (ALIC, VS, medial STN) and medical centers suggested optimal white matter streamlines are shared amongst several nuclei targets (20, 23). However, a large body of work suggests each stimulation site elicits distinct clinical effects by engaging specific circuits (68). Small studies comparing ventral-capsule/ventral-striatum (VC/VS) and STN DBS for OCD suggest that while both sites reduce YBOCS scores, they may do so via different mechanisms; VC/VS DBS was associated with improved mood by MADRS clinical scales whereas STN DBS improved cognitive flexibility by a set-shifting task (69). Further research into the underlying mechanism of NAc DBS for OCD revealed a decrease in NAc-mPFC connectivity (similar to imaging findings in capsulotomy) and provocation-related low-frequency frontal EEG oscillations (56). Local field potential (LFP) recordings from ventral STN DBS during DBS surgeries revealed low-frequency theta oscillations (typically associated with cognitive control and decision making), the power of which inversely related with YBOCS severity (70–72). While VS/NAc DBS may exert its effects via reducing excessive avoidance, STN DBS may provide relief by interrupting pathologic errors in action selection (73). Thus, DBS in distinct regions appears to modulate pathophysiology in cognitive and affective domains.

DBS provides the potential for closed-loop, or adaptive, neuromodulation to precisely treat OCD symptoms. Intracranial recordings of patients with OCD experiencing compulsions typically demonstrate increases in low-frequency oscillation power (delta: 1–4 Hz) across basal ganglia (globus pallidus *pars externa* – GPe, nucleus accumbens – NAc) structures (74). Current efforts by our group for personalized closed-loop DBS for OCD demonstrated that DBS guided by low-frequency oscillations tied to symptom provocation can reliably ameliorate symptoms in a patient (25). However, the dynamics of core obsessive-compulsive symptoms to prefrontal inhibitory control and reward sensitivity (which may interact differently in OCD subtypes) is unknown (75, 76). Recent work investigating LFPs from several deep structures in patients with OCD revealed increases in delta and alpha (8–12 Hz) power across GPe, NAc, and ALIC during provocation across OCD subtypes, suggesting a core obsessive-compulsive marker (74). In addition, high frequency activity (30–90 Hz) in OFC correlates with OCD symptom scores, and is suppressed by VC stimulation (77).

However, the interaction of these markers and the relative symptom burden of harm avoidance vs incompleteness remains unknown. To better personalize symptom biomarkers and DBS targets in patients with intractable OCD, ongoing studies incorporate clinical and behavioral testing while patients undergo inpatient stereo-EEG monitoring and stimulation at various sites (VS, STN, cingulate) (78) in a similar fashion to prior work with personalized DBS for depression (79). As clinical trials of DBS for depression have advanced from an anatomically- to a connectivity-defined target in the subcallosal cingulate (80, 81), ideally DBS for OCD will also incorporate circuit-based considerations for stimulation sites, along with functional symptom correlates (82).

### 4.2 Transcranial magnetic stimulation

The invasiveness of neurosurgical techniques such as lesioning and DBS inherently limit patient access and scalability for the treatment of OCD, which has led to increased interest in non-invasive means of circuit engagement such as TMS (relevant studies listed in **Table 4**). TMS uses repetitive magnetic pulses to modulate neuronal activity, with different parameters of repetitive stimulation (rTMS), including theta burst stimulation (TBS) shown to induce long term potentiation (LTP) and long term depression (LTD)-like effects (83). TMS is most commonly used for the treatment of depression, especially in treatment-resistant patients, an indication for which it has been FDA approved since 2008 (84). Major advantages of TMS are that it is non-invasive and has minimal side effects. However, unlike the other modalities described in this review, TMS is unique in that it is only able to reach cortical targets, being unable to modulate deeper than 4–6 cm from the scalp even with “deep TMS” coils (85). The ability to noninvasively target brain regions allows for TMS to be utilized both therapeutically and as a mechanism to investigate neural circuits. There are several cortical targets associated with OCD which have been investigated with TMS (with FDA approval in 2018 for deep TMS of the dorsomedial PFC/dmPFC and ACC) (86, 87).

**Table 4:** TMS studies for OCD across cortical targets and protocols. MT = motor threshold.

Study	Design	Target/protocol	N	Response	Main findings
Carmi 2019 (99)	Multi-center (11 sites) double-blind sham-controlled RCT	Deep TMS, H7 coil over dmPFC/ACC; 20 Hz; daily × 6 week safter individualized symptom provocation	99	38.1% vs 11.1% response (active vs sham) at end; 45.2% vs 17.8% at 1-mo	Led to FDA clearance (2018) of H7-coil dTMS for OCD
Roth 2021 (100)	Naturalistic post-marketing data, 22 clinical sites	dmPFC/ACC dTMS, HF protocol (50 trains of 2s duration, inter-train interval 20s, 2000 pulses/session) ± iTBS (3 pulses at 50 Hz, 5 Hz burst frequency, 2s ON/8s OFF, 1800 pulses/session)	219	52.9% sustained (>1 mo) response	Reproduces pivotal trial in routine practice
Mantovani 2010 (103)	Sham-controlled RCT	Bilateral SMA, 1 Hz, 100% MT, 1200 pulses/day, 4 weeks	21	67% vs 22% response in completers; 25% vs 12% YBOCS reduction	Active SMA rTMS normalized motor-cortex hemispheric asymmetry of motor threshold
Nauczyciel 2014 (101)	Double-blind crossover (1-wk × 2, 1-mo washout)	Right OFC via double-cone coil, low-frequency rTMS 1 Hz, 120% MT, 1200 pulses/session	22	YBOCS −6 vs −2 (active vs sham), p = 0.07	Active stim ↓ bilateral OFC metabolism on PET; OFC accessible noninvasively
Williams 2021 (105)	Open-label accelerated pilot	Modified cTBS (cTBSmod), fMRI-guided right frontal pole, 1800 pulses/session, 3 pulses/burst at 30 Hz, repeated at 60 Hz, 10 sessions/day, 90% MT	7	57% response at Day 14; 71% at any time point	Accelerated, high-dose cTBSmod feasible; only transient headache/fatigue
Rostami 2020 (106)	Retrospective	rTMS to dlPFC or SMA (1 Hz, 1800 pulses/session, 20 sessions)	65	46.2% response (≥30% YBOCS)	No significant target difference
Baldi 2024 (108)	Proof-of-concept	CTBS TMS (50 Hz triplets of pulses, triplets delivered at 5 Hz for 60 s, 100% MT) to fMRI-defined cortical site most connected to STN (and STN+NAc)	9	Connectivity changes at deep targets within 5–25 min	Personalized fMRI-guided targeting reaches deep circuits
Fitzsimmons 2025 (109)	Comparative	rTMS (10 Hz, 110% MT, 30, 30 x 10-sec trains, 3000 pulses/session) to dlPFC (Tower of London) vs preSMA (stop-signal)	61	Similar YBOCS improvement	dlPFC rTMS ↓ planning-related BOLD correlating with response

The dorsolateral prefrontal cortex (dlPFC) is the most commonly used target for major depressive disorder and has been investigated due to its role in goal-directed behavior, inhibitory control, and executive functioning with OCD (88, 89). Thus, modulation of the dorsal cognitive circuit leading to improved top-down emotional regulation underlies the clinical effect of dlPFC TMS. The dlPFC is functionally connected with subcortical areas such as the caudate nucleus (90), and the degree of connectivity between these prefrontal areas and deep structures has been shown to correlate with symptom alleviation (56, 91). LTP-like rTMS of the left dlPFC and LTD-like rTMS of the right dlPFC have both been shown to have therapeutic effects, with a meta-analysis of 21 randomized controlled trials demonstrating significantly greater improvement in YBOCS (compared to sham, Hedges’ *g* = -0.502, [95%CI = -0.708, -0.296]) in the treatment of OCD (92, 93).

Dorsomedial prefrontal cortex (dmPFC) has been implicated in OCD as a core mediator of inhibitory control and response adaptation, and like the dlPFC is functionally connected to the wider subcortical OCD network (94). This region is also proximal to the anterior cingulate cortex (ACC), which is functionally connected to the ventral striatum (13). Although considered a subcortical structure, the ACC is able to be stimulated at its ventral aspect by a “deep TMS” H7 coil. Studies comparing Stroop-related functional MRI activity found decreased caudate activity after clinically effective deep TMS to ACC/mPFC, suggesting clinical improvement also involves the dorsal cognitive circuit (95). ACC or mPFC TMS may also affect dysregulated emotional responses to uncertainty in the fronto-limbic circuit (specifically, PFC-amygdala hyperactivity) as evidenced by several studies (96–98). Pilot data investigating stimulation of this area led to a pivotal clinical trial in 2019 (99) which showed clinically significant reduction in symptoms and led to subsequent FDA approval of this protocol for treatment. The efficacy of stimulating dmPFC has borne out in real-world clinical practice with sustained (> 1 month) response rates of 52.9% (100).

Other cortical areas that have been evaluated for OCD include the orbitofrontal cortex (OFC) and the supplemental motor area (SMA). The OFC, similar to the ACC, is thought to be involved in the ventral affective circuit and is functionally connected to the ventral striatum (13). This area has been evaluated by TMS and found to be efficacious for symptom relief with an LTD-like stimulation pattern (101). It is worth noting that due to the proximity of this area to the lower forehead and eye, participants may have discomfort with stimulation at this site, somewhat limiting widespread use. The SMA is part of the sensorimotor OCD circuit and dysfunction is thought to be related to motoric and cognitive inflexibility common in OCD. It is functionally connected to subcortical structures including the striatum and circuit of Papez (102). The SMA has been targeted efficaciously with LTD-like stimulation, typically delivered bilaterally (103).

Early TMS research often involved shorter protocols, limiting generalizability to today’s practice as a modern course of TMS involves 25–30 treatments. Typically treatments are given once daily; however, one major area of recent innovation in the delivery of TMS has been the adoption of accelerated protocols. This was initially shown safe and efficacious for depression, with the landmark SAINT protocol showing a 90.5% remission rate over the course of only five days (104). This paradigm is now being investigated for OCD as well; Williams et al. (2021) conducted a pilot study delivering modified continuous TBS (cTBSmod) to seven patients targeting the right frontal pole (which overlaps the OFC) and showed that accelerated TMS was well tolerated with a 57% response rate in OCD symptoms as measured by the YBOCS (105).

As with modalities described, there is evidence suggesting that tailoring TMS targets to various symptom profiles can provide better personalized treatment for OCD. Rostami et al. (2020) conducted a retrospective study on 65 individuals who received rTMS targeted at the DLPFC or SMA due to elevated depressive symptoms in addition to core OCD symptoms (106). Although this retrospective study found no statistical difference in OCD symptom reduction between treatment at the two different brain regions, there was a reported 46.2% response rate as defined by the criteria of a 30% reduction in Y-BOCS scores (106). Intriguingly, a separate meta-analysis of dlPFC and preSMA rTMS for OCD found greater effect sizes for obsessive versus compulsive symptoms (107). Larger network meta-analyses including 21 studies with 662 patients found that rTMS is efficacious across several targets (OFC, dlPFC, mPFC, ACC, preSMA) (93). Within these targets, optimal stimulation parameters include high-frequency TMS to bilateral dlPFC, low-frequency TMS to bilateral preSMA, and low-frequency TMS to right dlLPFC.

Another emerging innovation is in tailoring TMS targets based on functional neuroimaging, cognitive tasks, and individualized phenotypes. Baldi et al. (2024) conducted a proof-of-concept study that utilized functional neuroimaging to identify the cortical area with greatest connectivity to the STN and the cortical area most connected to both the STN and NAc and applied TMS at these identified regions. Significant changes in functional connectivity at these deep areas with cortical stimulation were seen as early as 5 minutes after TMS, with more lasting effects observed around 25 minutes (108). More recent trials have generated TMS targets based on task-based fMRI activation from the Tower of London task in the dlPFC and the stop-signal task in the preSMA (109). Although patients demonstrated similar clinical improvement, DLPFC rTMS elicited decreases in planning-related BOLD activity which correlated with clinical improvement. On the other hand, preSMA rTMS did not elicit consistent changes in BOLD activity associated with inhibition or error processing. Future studies combining intracranial recordings and TMS may be better suited to probe the interaction of tasks, symptom domains, and circuits.

### 4.3 Focused ultrasound

The ideal modulatory therapy for OCD would be noninvasive and with few side effects like TMS, but able to target deep targets like DBS. Focused ultrasound therapy (FUS) is a rapidly developing modality which offers this combination. There are two forms of FUS being investigated for OCD; magnetic resonance-guided high-intensity focused ultrasound (MRgFUS) and low-intensity focused ultrasound (LIFU) (110). MRgFUS is an ablative therapy in which convergent ultrasonic waves are focused to create tissue heating and resultant coagulative necrosis. MRgFUS is highly precise with millimeter accuracy, and is FDA-approved for the treatment of essential tremor and Parkinson’s disease (111). Applications for OCD have primarily focused on recapitulating surgical lesions/procedures (such as cingulotomy, capsulotomy) using high-intensity focused ultrasound (112). Specifically, an open-label trial of MRgFUS capsulotomy in 15 patients with OCD demonstrated clinically significant reductions of YBOCS scores at 6 (23%) and 12 (35%) months (113). A separate 10-year 11-patient cohort receiving MRgFUS capsulotomy demonstrated YBOCS response rates at 2 (55%) and 10 (64%) years post-capsulotomy (114).

LIFU, conversely, is a non-ablative therapy which is performed at orders of magnitude less intensity than MRgFUS and can be performed either in-scanner or in-clinic using real-time neuronavigation. LIFU has been shown to modulate tissue in a reversible fashion, similar to other noninvasive therapies such as TMS (115), although with an advantage over TMS in that it is not limited to cortical targets and is capable of sonication at least 4–8 cm deep to the scalp. Initial LIFU studies investigating its application to VC/VS in healthy individuals suggest that LIFU-associated alterations in VC/VS-OFC and -ACC connectivity could lead to potential applicability to treating patients with OCD (116). LIFU of the ALIC has similarly also been investigated for depression with resultant changes in connectivity, and a sham-controlled pilot study showed rapid improvement in symptoms when directed to the cingulum for the treatment of depression (117, 118). Although clinical studies are in preliminary stages, LIFU may allow for non-invasive and reversible neuromodulation of deep nuclei structures.

### 4.4 Other modalities

Several other neuromodulation modalities, albeit with small evidence bases, have been investigated for OCD. Electroconvulsive therapy (ECT), arguably the original neuromodulation therapy, delivers an electric charge through scalp electrodes under brief general anesthesia in order to induce a seizure (119). While rapidly effective for mood or psychotic episodes, its role in OCD is less certain. Furthermore, its nonspecific mechanism of action makes circuit-based manipulation difficult. Nevertheless a systematic review examining the effectiveness of ECT found no randomized control trials, but noted 60% of examined cases reported some improvement (with no clear response criteria) in OCD symptoms (120).

Another promising noninvasive neuromodulation modality is transcranial direct current stimulation (tDCS). Like ECT it involves passing low current through two scalp electrodes to affect the excitability of underlying cortex (121). Briefly, anodal tDCS increases cortical excitability via membrane depolarization, and cathodal tDCS decreases excitability via hyperpolarization. A meta-analysis of 10 randomized sham-controlled trials of tDCS for OCD found a significant reduction in YBOCS (standardized mean difference: -0.56 [95%CI = -0.87, -0.26] across several anatomic sites and cathodal/anodal configurations (122), The largest of these trials involved 43 patients receiving 20 consecutive daily sessions of cathodal or sham tDCS over the pre-SMA, with cathodal tDCS significantly reducing YBOCS compared to sham (Cohen’s *d*: 0.62 [95%CI = 0.06, 1.18] (123). Like TMS, inhibitory stimulation of the pre-SMA may work to reduce its hyperactivity observed in patients with OCD, potentially facilitating more adaptive response inhibition in the sensorimotor circuit (124). A separate single-blinded trial administered two weeks of cathodal tDCS to 18 patients using either cerebellum-OFC, pre-SMA, or OFC-pre-SMA montages (125). After stimulation YBOCS was significantly reduced up to 20% after cerebellum-OFC and OFC-pre-SMA tDCS, and this reduction persisted up to one month. These clinical effects were also accompanied by dissociable alterations in scalp EEG beta-frequency coherence (decreased dACC-inferior parietal lobule coherence with cerebellum-OFC tDCS, increased bilateral superior frontal gyri coherence with OFC-pre-SMA tDCS). Taken together these results demonstrate the potential for circuit-specific neuromodulation with different tDCS montages.

## 5 Discussion

OCD is a common but disabling psychiatric disorder. While the neuromodulation modalities reviewed here demonstrate promise for symptom relief, much work remains to be done on tailoring stimulation strategies in a patient- and symptom-specific manner. This could be accomplished through various complementary means, whether by better phenotyping patients to guide interventional target, or by using closed-loop deep brain stimulation to deliver stimulation in response to a symptom-specific biomarker (22, 25). In any case, matching efforts must also be made to improve access to these treatments, as traditional inequities in mental health parity remain persistent (54).

When reviewing all the discussed invasive modalities in a clinical context (**Table 5**), lesional procedures (cingulotomy and capsulotomy) offer durable benefit with response rates of roughly 50–78%, but they are irreversible. DBS can match lesional efficacy (response rates up to 66%) while adding reversibility, programmability, and the potential closed-loop personalization, at the cost of invasiveness, expense, and intensive follow-up. While LIFU presents the potential for non-invasive/non-ablative modulation of deep structures/circuits, its evidence remains preliminary. When considering non-invasive cortical targets, TMS has demonstrated the largest evidence base, with emerging accelerated and fMRI-guided protocols, but it cannot reach deeper than 4–6 cm and requires 25–30 sessions. tDCS may provide a less expensive alternative to TMS, but its evidence also remains limited. And although ECT is the good standard for acute mood episodes, it has no RCTs and a nonspecific mechanism that resists circuit-level targeting.

**Table 5:** Comparative summary of the clinical advantages and limitations of each neuromodulation modality discussed in this review..

Modality	Pros	Cons
Cingulotomy	~56% durable improvement; no clear long-term neuropsych deficits	Irreversible; reduced behavioral spontaneity
Capsulotomy	48-78% response across techniques; ventral/gamma-knife/laser variants reduce side effects	Irreversible; apathy & executive dysfunction (esp. dorsal lesions); ~13% weight gain
Deep Brain Stimulation	Reversible, programmable; up to 66% response; circuit-specific targeting; closed-loop capable	Invasive; expensive; intensive follow-up; optimal target unsettled
TMS	Noninvasive; FDA-approved (dmPFC/ACC); efficacious across multiple cortical targets; accelerated & fMRI-guided protocols emerging	Limited to ~4–6 cm depth; 25–30 sessions; OFC TMS uncomfortable
MRgFUS	Incisionless, millimeter precision; durable capsulotomy (~55% at 2 yr, ~64% at 10 yr)	Ablative/irreversible; small, largely open-label OCD evidence
LIFU	Noninvasive, reversible; reaches deep targets (4–8 cm)	Preliminary OCD data; dosing/targeting unsettled
ECT	Rapidly effective for comorbid mood/psychotic features; long safety record	No OCD RCTs; nonspecific mechanism; cognitive side effects
tDCS	Cheap, portable, noninvasive; meta-analytic efficacy (cathodal pre-SMA)	Cortex only; modest effect sizes; protocols not standardized

Overall, advances in understanding of cognitive neuroscience and OCD pathophysiology point towards an interaction of multiple dorsal/cognitive and ventral/affective frontostriatal circuits. While neurosurgical lesioning approaches such as cingulotomy and capsulotomy have allowed for coarse disentangling of these circuit functions, modern neuromodulation modalities like TMS and DBS allow for circuit-specific manipulation. Looking forward, advances such as focused ultrasound promise precise non-invasive modulation of deep targets while closed-loop DBS allows for incorporating spatio-temporal circuit interactions and the development of adaptive patient- and symptom-specific treatments.
